# Deciphering the functional diversity of the gut microbiota of the black soldier fly (*Hermetia illucens*): recent advances and future challenges

**DOI:** 10.1186/s42523-023-00261-9

**Published:** 2023-08-31

**Authors:** Maurielle Eke, Kévin Tougeron, Alisa Hamidovic, Leonard S. Ngamo Tinkeu, Thierry Hance, François Renoz

**Affiliations:** 1 Biodiversity Research Centre, Earth and Life Institute, UCLouvain, 1348, Louvain-la-Neuve, Belgium; 2 Department of Biological Sciences, University of Ngaoundéré, PO BOX 454, Ngaoundéré, Cameroon; 3 UMR CNRS 7058 EDYSAN (Ecologie et Dynamique des Systèmes Anthropisés), Université de Picardie Jules Verne, Amiens, 80039 France; 4 Research Institute in Bioscience, Université de Mons, Mons, 7000 Belgium; 5 Institute of Agrobiological Sciences, National Agriculture and Food Research Organization (NARO), Tsukuba, 305-8634 Japan

**Keywords:** Mycobiota, Virobiota, Metabarcoding, Symbiosis, Organic waste, Bioconversion, Industrial rearing

## Abstract

Bioconversion using insects is a promising strategy to convert organic waste (catering leftovers, harvest waste, food processing byproducts, etc.) into biomass that can be used for multiple applications, turned into high added-value products, and address environmental, societal and economic concerns. Due to its ability to feed on a tremendous variety of organic wastes, the black soldier fly (*Hermetia illucens*) has recently emerged as a promising insect for bioconversion of organic wastes on an industrial scale. A growing number of studies have highlighted the pivotal role of the gut microbiota in the performance and health of this insect species. This review aims to provide a critical overview of current knowledge regarding the functional diversity of the gut microbiota of *H. illucens*, highlighting its importance for bioconversion, food safety and the development of new biotechnological tools. After providing an overview of the different strategies that have been used to outline the microbial communities of *H. illucens*, we discuss the diversity of these gut microbes and the beneficial services they can provide to their insect host. Emphasis is placed on technical strategies and aspects of host biology that require special attention in the near future of research. We also argue that the singular digestive capabilities and complex gut microbiota of *H. illucens* make this insect species a valuable model for addressing fundamental questions regarding the interactions that insects have evolved with microorganisms. By proposing new avenues of research, this review aims to stimulate research on the microbiota of a promising insect to address the challenges of bioconversion, but also fundamental questions regarding bacterial symbiosis in insects.

## Introduction

The increase in food demand due to the growth of the world’s population generates huge amounts of organic waste (food waste, livestock products, etc.) that have adverse environmental, social and economic consequences [1]. One of the strategies to manage this waste is to integrate it into a sustainable circular system and convert it into valuable biomass [2]. In this context, bioconversion using insect larvae is considered a promising technique for converting organic waste into biomass that can be used for multiple purposes, including as a source of proteins and lipids for animal feed and human food, pharmaceuticals, biofuels, lubricants, biogas, and fertilizers [3–8]. Insect bioconversion is gaining momentum as a research topic and commercial opportunity [9]. Although promising, insect bioconversion is still in its infancy: only a few insect species are currently used commercially for the bioconversion of organic waste and the underlying mechanisms remain largely unknown [10, 11]. However, it has become clear that the gut microbes of these insects play a pivotal role in bioconversion processes [12].

The black soldier fly *Hermetia illucens* is currently the most widely used insect species for bioconversion of organic waste. The popularity of this insect species for bioconversion stems from its multiple advantages. One of the main advantages of this insect is that the larvae can grow rapidly on an astonishing variety of organic streams, including manure and agri-food waste [3, 13–15], allowing for the conversion of organic waste into biomass in the form of protein-rich (40–45%, dry basis) and lipid-rich (35–40%, dry basis) larvae that can be used to generate biofuels and as protein source for livestock and aquaculture feed [16–25]. The advantages of *H. illucens* for bioconversion of organic waste are in line with the sustainable circular economy policy adopted in many countries around the world [3], although rearing conditions and legislation for the mass use of this insect still needs to be adapted for a successful industrial sector to emerge [9]. As with any farm animal, identifying the optimal rearing conditions for *H. illucens* is necessary to improve productivity, and in recent years many studies have focused on identifying the parameters that affect productivity under mass rearing conditions [26–31]. These parameters include abiotic factors such as thermal and lighting conditions, humidity and nutritional value of the consumed substrate, but also biotic factors such as the microbial flora colonizing the rearing substrate and the digestive tract of *H. illucens*.

The microbiota is an increasingly important research topic in the field of biology and medicine. It represents the set of microorganisms (bacteria, fungi, viruses, etc.) interacting with a living organism, which can be commensal, mutualist or pathogenic [32]. The gut microbiota of *H. illucens* has recently received particular attention first because, as in many other insect and animal species, it can contribute significantly to the performance and health of the insect (e.g., by assisting the insect in the digestion of certain organic compounds) and thus represents a key dimension to consider in the optimization of *H. illucens* mass rearing and bioconversion processes [33]. The study of the *H. illucens* gut microbiota is also motivated by food safety aspects, as it is essential to determine if these insects potentially dedicated to feed and food can be carriers of pathogenic microorganisms that can endanger animal and human health [34]. However, beyond these applied aspects, the study of the *H. illucens* microbiota also offers the opportunity to address fundamental issues regarding the nature of the interactions between insects and microorganisms. These include understanding the specific adaptations that allow this insect to cope with the myriad of pathogenic microbes that thrive in the various organic substrates on which it feeds. These adaptive capacities may include a robust immune system, a strong intestinal epithelial-digestive barrier, the ability to produce antimicrobial compounds and association with a protective microbiota [32]. Another fundamental issue regarding this insect species is the role played by the gut microbiota in the conversion of very diverse and sometimes nutrient-poor organic matter into high quality nutrients. Although we now know that the *H. illucens* gut microbiota includes a diversity of microbial associates [35], their role in digestive processes and how they function collectively and with the host remain unknown. Finally, the determination of the parameters, in particular the rearing conditions (type of substrate, thermal conditions, etc.), that influence the structuring and functioning of microbial communities associated with the *H. illucens* gut is an important issue in an applied context.

Over a few years, *H. illucens* has become one of the most studied insect species, with an increasing interest in its gut microbiota. However, despite numerous publications in recent years, the study of the microbiological dimension of this insect remains sketchy. This review aims to provide a critical overview of current knowledge regarding the functional diversity of the gut microbiota of *H. illucens*, highlighting its importance for practical applications in the recycling and bioconversion of organic waste, but also to answer more fundamental research questions raised by the singular lifestyle of this insect. Each section addresses specific aspects of the *H. illucens* microbiota and aims to pinpoint research gaps that need to be filled to obtain a more accurate picture of its functional diversity. Emphasis is given to technical strategies and aspects of host biology that require attention in the near future of research. By proposing new avenues of research, this review aims to stimulate research on the microbiota of a promising insect to address the applied and industrial challenges of bioconversion, but also fundamental questions regarding the interaction between insects and their gut microbes.

## Approaches used to define the composition of the *H. illucens* gut microbiota

Over the past two decades, a new generation of sequencing technologies has emerged that can provide previously unattainable information about the alpha and beta diversity of all domains of life, particularly in the field of microbiology [36]. The metabarcoding approach, which involves the use of polymerase chain reaction (PCR) and high-throughput sequencing (HTS) to assess the microbial diversity of organisms in a sample, can now rapidly provide a comprehensive picture of the taxonomic composition of microbial communities associated with animals, including insects [37]. The composition of the microbiota associated with *H. illucens* residing in its gut has been addressed in numerous recent studies. From 2011 to early 2023, a total of 42 publications have documented the composition of the microbiota of this insect species using high throughput approaches (Tables 1, 2 and 3), with particular interest in the impact of food substrate on the diversity of bacterial communities present in the larval gut using the 16 S rRNA gene [30, 38–56]. Microbial communities associated with *H. illucens* were first examined by pyrosequencing technology [38, 57–59] and then mostly by Illumina sequencing [30, 39–44, 47, 48, 50, 52–56, 60–75]. Illumina sequencing has the advantage of being relatively affordable but generates short reads (approximately 400 bp) that provide limited resolution for identifying microbial taxa and conducting phylogenetic approaches to infer evolutionary relationships and predict functional profiles [76, 77]. More recently, Oxford Nanopore MinION sequencing has been used to address the bacterial diversity of *H. illucens* [49], but the use of this approach is still limited. By generating long reads (approximately 1,500 bp), this and other long-read sequencing approaches allow for better taxonomic assignment of insect-associated microorganisms and, as they become more mainstream, should provide a more accurate picture of the *H. illucens* microbiota [76, 78].

**Table 1:** Published studies addressing the taxonomic composition of the bacterial microbiota associated with *H. illucens*

References	Rearing conditions	Substrates	Life stage	Tissue	Methodology
Jeon et al. 2011 [38]	Not specified	Food waste; calf forage; cooked rice	Larvae	Whole gut	Pyrosequencing
Zheng et al. 2013 [57]	27 °C RH: Ambient	Gainesville diet	Eggs, larvae, prepupae, pupae, adults	Whole individual	Pyrosequencing
Zheng et al. 2013 [58]	27 °C RH: Ambient	Gainesville diet	Eggs and larvae	Whole individual	Pyrosequencing; cultivation followed by partial 16 S rRNA gene sequencing
Cai et al. 2018 [60]	27 °C RH: 70%	Chicken manure	Larvae	Whole gut	Illumina HiSeq amplicon sequencing
Cai et al. 2018 [61]	27.5 °C RH: 70%	Wheat bran	Larvae	Whole gut	Illumina HiSeq amplicon sequencing; cultivation followed by partial 16 S rRNA gene sequencing
Bruno et al. 2019 [39]	27 °C RH: 70%	Mixture of vegetable flours; vegetables; fish meal	Larvae	Midgut (anterior, middle, and posterior regions)	Illumina MiSeq amplicon sequencing
Jiang et al. 2019 [30]	Not specified	Raw food waste	Larvae	Whole gut	Illumina HiSeq amplicon sequencing
Zhan et al. 2019 [41]	25 °C 35–40%	Food waste; poultry manure; dairy manure; swine manure	Larvae	Midgut	Illumina HiSeq amplicon sequencing
Wynants et al. 2019 [40]	24.9–29.1 °C RH: 21–71%	Fruit/vegetable waste; supermarket and restaurant waste; poultry blood; poultry manure; chicken feed; wheat bran; wheat flour; yeast concentrate; brewer’s spent grains	Larvae	Whole individual	Illumina MiSeq amplicon sequencing
Ao et al. 2020 [43]	27.5 °C RH: 70%	Mixture of wheat flour and bran; swine/chicken manure	Larvae	Whole gut	Illumina MiSeq amplicon sequencing
Callegari et al. 2020 [80]	25 °C RH: 60–65%	Mixture of wheat germ, alfalfa and corn flour	Larvae	Whole gut	Cultivation followed by partial 16 S rRNA gene sequencing
Cifuentes et al. 2020 [62]	25 °C RH: Not specified	Commercial chicken feed	Larvae and prepupae	Whole gut	Illumina MiSeq amplicon sequencing
Khamis et al. 2020 [63]	Not specified	Not specified	Larvae	Whole individual	Illumina MiSeq amplicon sequencing
Klammsteiner et al. 2020 [42]	27 °C RH: 60%	Chicken feed; freshly cut grass; vegetables	Larvae	Whole gut	Illumina MiSeq amplicon sequencing
Liu at al. 2020 [64]	29 °C RH: Not specified	Soya meal	Larvae	Whole gut	Illumina MiSeq amplicon sequencing
Raimondi et al. 2020 [65]	20–33 °C RH: 65%	Mixture of vegetable flours	Larvae and prepupae	Whole individual	Illumina MiSeq amplicon sequencing; cultivation followed by identification on specific Agar plates
Shelomi et al. 2020 [54]	Not specified	Postproduction soy pulp; postconsumer cafeteria waste	Larvae	Whole gut	Illumina HiSeq amplicon sequencing; cultivation followed by partial 16 S rRNA gene sequencing
Wu et al. 2020 [74]	30 °C RH: Not specified	Wheat bran	Larvae	Whole gut	Illumina MiSeq amplicon sequencing
Galassi et al. 2021 [55]	25 °C RH: 60%	Hen diet; okara; maize distillers; brewer’s grains	Larvae	Whole gut	Illumina MiSeq amplicon sequencing
Tegtmeier et al. 2021 [44]	27 °C RH: 65%	Chicken feed; cottonseed press cake	Larvae	Whole gut	Illumina MiSeq amplicon sequencing
Tegtmeier et al. 2021 [66]	27 °C RH: 65%	Chicken feed	Larvae	Whole gut	Illumina MiSeq amplicon sequencing; cultivation followed by partial 16 S rRNA gene sequencing
Gorrens et al. 2021 [45]	27 °C RH: 60%	Chicken feed; fiber-rich ingredients	Larvae	Whole individual	Cultivation followed by identification with mass spectrometry
Greenwood et al. 2021 [46]	30 °C RH: 64%	Grain-based feed; mixture of brewer’s spent grain and a plant-based sweetener; vegetable waste stream	Larvae	Whole gut	Ion Torrent sequencing
Klammsteiner et al. 2021 [47]	27 °C RH: 60%	Chicken feed; food waste; oil wast	Larvae	Whole gut	Illumina MiSeq amplicon sequencing
Li et al. 2021 [73]	28 °C RH: 60–70%	Food waste	Larvae	Whole gut	Illumina MiSeq amplicon sequencing; cultivation followed by partial 16 S rRNA gene sequencing
Liu et al. 2021 [67]	29 °C RH: 65%	Soya meal; oxytetracycline bacterial residue	Larvae	Whole gut	Illumina MiSeq amplicon sequencing
Osimani et al. 2021 [48]	27 °C RH: 65%	Mixtures of coffee by-products and microalgae	Larvae	Whole individual	Illumina MiSeq amplicon sequencing
Shumo et al. 2021 [49]	28 °C RH: 65%	Chicken manure; kitchen waste	Larvae	Whole individual	Oxford Nanopore MinION Sequencing; cultivation followed by partial 16 S rRNA gene sequencing
Tanga et al. 2021 [50]	Ambient conditions	Brewers’ spent grains; kitchen food waste; poultry manure; rabbit manure	Larvae	Whole gut	Illumina MiSeq amplicon sequencing
Yang et al. 2021 [53]	27 °C RH: 70%	Gainesville diet; starvation	Larvae	Whole individual	Illumina HiSeq amplicon sequencing
Zhang et al. 2021 [68]	30 °C RH: Not specified	Chicken manure	Larvae	Whole gut	Illumina MiSeq amplicon sequencing
Zhineng et al. 2021 [51]	25 °C RH: 60–70%	Wheat bran + soybean powder; food waste	Larvae	Whole gut	Metagenomic sequencing (approach not specified)
Soomro et al. 2021 [56]	27 °C RH: 70%	Corn flour + bran	Larvae	Whole individual	Illumina HiSeq amplicon sequencing
Cifuentes et al. 2022 [79]	25 °C RH: Not specified	Chicken feed	Larvae	Whole gut	Cultivation followed by partial 16 S rRNA gene sequencing
Gorrens et al. 2022 [52]	30 °C / ambient temperature RH: 50–60%	Chiken feed; mixture of vegetable coproducts; pig feed; pig feed + wheat bran	Larvae	Whole individual	Illumina MiSeq amplicon sequencing
Pei at al. 2022 [69]	30 °C RH: 70%	Wheat bran; food waste + peanut shell powder	Larvae	Whole gut	Illumina MiSeq amplicon sequencing
Querejeta et al. 2022 [70]	Not specified	Gainesville diet	Eggs, larvae, prepupae, pupae, adults	Whole gut	Illumina MiSeq amplicon sequencing
Zhang et al. 2022 [72]	27 °C RH: 60–70%	Pig manure	Larvae	Whole gut	Illumina MiSeq amplicon sequencing; cultivation followed by partial 16 S rRNA gene sequencing
Yu et al. 2023 [71]	28 °C RH: Not specified	Artificial diet for *Drosophila melanogaster*	Larvae	Whole gut	Illumina MiSeq amplicon sequencing

**Table 2:** Summary of the taxonomic composition of the bacterial microbiota associated with *H. illucens*

References	Life stage	Substrates	Dominant identified phyla	Dominant bacterial genera
Jeon et al. 2011 [38]	Larvae	Food waste; calf forage; cooked rice	Actinomycetota, Bacteroidota, Bacillota, Fusobacteria and Pseudomonadota	*Citrobacter, Enterococcus, Klebsiella, Leminorella, Morganella* and *Providencia*
Zheng et al. 2013 [57]	Eggs, larvae, prepupae, pupae, adults	Gainesville diet	Acidobacteria, Verrucomicrobia, Bacillota, Actinomycetota, Pseudomonadota and Bacteroidota	*Providencia, Bacteroides, Sphyngobacterium, Dysgonomonas and Sanguibacter*
Zheng et al. 2013 [58]	Eggs and larvae	Gainesville diet	Bacillota, Actinomycetota, Bacteroidota and Pseudomonadota	*Bacillus, Cellulomonas, Empedobacter, Enterobacter, Gordonia, Kurthia, Microbacterium* and *Micrococcus*
Cai et al. 2018 [60]	Larvae	Chicken manure	Bacillota, Pseudomonadota, Bacteroidota and Actinomycetota	*Providencia, Enterococcus, Morganella and Dysgonomonas*
Cai et al. 2018 [61]	Larvae	Wheat bran	Bacillota, Pseudomonadota, Bacteroidota, Actinomycetota and Fusobacteriota	*Flavisolibacter, Proteus, Klebsiella, Actinomyces, Globicatella, Providencia, Enterococcus* and *Ignatzschineria*
Bruno et al. 2019 [39]	Larvae	Mixture of vegetable flours; vegetables; fish meal	Actinomycetota, Bacillota, Bacteroidota and Pseudomonadota	*Dysgonomonas, Providencia, Blautia, Shingobacterium, Morganella* and *Bacillus*
Jiang et al. 2019 [30]	Larvae	Raw food waste	Bacillota, Bacteroidota and Pseudomonadota	*Bacillus, Lactobacillus, Dysgonomonas, Enterococcus* and *Providencia*
Zhan et al. 2019 [41]	Larvae	Food waste; poultry manure; dairy manure; swine manure	Bacillota, Bacteroidota and Pseudomonadota	Not specified
Wynants et al. 2019 [40]	Larvae	Fruit/vegetable waste; supermarket and restaurant waste; poultry blood; poultry manure; chicken feed; wheat bran; wheat flour; yeast concentrate; brewer’s spent grains	Pseudomonadota and Bacillota	*Morganella, Bacillus, Enterococcus, Providencia* and *Lactobacillus*
Ao et al. 2020 [43]	Larvae	Mixture of wheat flour and bran; swine/chicken manure	Bacillota, Bacteroidota, Actinomycetota and Pseudomonadota	*Enterococcus, Providencia, Morganella, Klebsiella, Ignatzschineria* and *Clostridium*
Callegari et al. 2020 [80]	Larvae	Mixture of wheat germ, alfalfa and corn flour	Pseudomonadota, Bacillota, Actinomycetota and Bacteroidota	*Providencia, Morganella, Klebsiella, Escherichia, Acinetobacter, Stenotrophomonas, Pseudomonas* and *Enterococcus*
Cifuentes et al. 2020 [62]	Larvae and prepupae	Commercial chicken feed	Bacillota, Bacteroidota, Actinomycetota and Pseudomonadota	*Morganella, Klebsiella, Providencia, Enterobacter, Enterococcus, Bacillus, Proteus, Actinomyces* and *Dysgonomonas*
Khamis et al. 2020 [63]	Larvae	Not specified	Bacillota, Bacteroidota, Actinomycetota and Pseudomonadota	*Ignatzschineria, Enterococcus, Dysgonomonas, Morganella, Providencia* and *Lactobacillus*
Klammsteiner et al. 2020 [42]	Larvae	Chicken feed; freshly cut grass; vegetables	Bacillota, Bacteroidota, Actinomycetota and Pseudomonadota	*Actinomyces, Dysgonomonas, Enterococcus* and *Morganella*
Liu at al. 2020 [64]	Larvae	Soya meal	Bacillota, Bacteroidota, Actinomycetota and Pseudomonadota	*Enterococcus*, *Ignatzschineria, Providencia* and *Morganella*,
Raimondi et al. 2020 [65]	Larvae and prepupae	Mixture of vegetable flours	Bacillota, Bacteroidota, Actinomycetota and Pseudomonadota	*Providencia, Klebsiella, Bacillus, Morganella, Alcaligenes, Bordetella* and *Kerstersia*
Shelomi et al. 2020 [54]	Larvae	Postproduction soy pulp; postconsumer cafeteria waste	Bacteroidota and Bacillota	*Bacillus, Citrobacter, Dysgonomonas, Porphyromonas* and *Parabacteroides*
Wu et al. 2020 [74]	Larvae	Wheat bran	Bacteroidota, Actinomycetota, Pseudomonadota and Bacillota	*Salana, Parabacteroidetes* and *Campylobacter*
Galassi et al. 2021 [55]	Larvae	Hen diet; okara; maize distillers; brewer’s grains	Bacteroidota, Actinomycetota, Pseudomonadota and Bacillota	*Providencia, Morganella* and *Klebsiella*
Tegtmeier et al. 2021 [44]	Larvae	Chicken feed; cottonseed press cake	Pseudomonadota and Bacillota	*Morganella, Providencia* and *Enterococcus*
Tegtmeier et al. 2021 [66]	Larvae	Chicken feed	Bacillota, Bacteroidota, Actinomycetota and Pseudomonadota	*Morganella, Enterococcus, Proteus, Providencia, Sphingobacterium, Klebsiella, Paenalcaligenes, Corynebacterium* and *Citrobacter*
Gorrens et al. 2021 [45]	Larvae	Chicken feed; fiber-rich ingredients	Bacillota and Pseudomonadota	*Escherichia, Klebsiella, Enterobacter, Enterococcus, Providencia* and *Morganella*
Greenwood et al. 2021 [46]	Larvae	Grain-based feed; mixture of brewer’s spent grain and a plant-based sweetener; vegetable waste stream	Bacillota, Bacteroidota, Actinomycetota and Pseudomonadota	*Morganella, Providencia, Lactobacillus, Enterococcus* and *Proteus.*
Klammsteiner et al. 2021 [47]	Larvae	Chicken feed; food waste; oil wast	Pseudomonadota, Bacteroidota, Bacillota and Actinomycetota	*Morganella, Providencia, Dysgonomonas* and *Lactobacillus*
Li et al. 2021 [73]	Larvae	Food waste	Pseudomonadota, Bacillota and Bacteroidota	*Ignatzschineria, Providencia, Proteus, Klebsiella* and *Vagococcus*
Liu et al. 2021 [67]	Larvae	Soya meal; oxytetracycline bacterial residue	Pseudomonadota, Bacillota and Actinomycetota	*Providencia, Enterococcus* and *Ignatzschineria*
Osimani et al. 2021 [48]	Larvae	Mixtures of coffee by-products and microalgae	Bacillota and Pseudomonadota	*Paenibacillus, Lysinibacillus, Solibacillus, Brevundominas, Morganella, Enterococcus* and *Paracoccus*
Shumo et al. 2021 [49]	Larvae	Chicken manure; kitchen waste	Pseudomonadota, Actinomycetota and Bacillota	*Providencia, Morganella, Brevibacterium, Staphylococcus* and *Bordetella*
Yang et al. 2021 [53]	Larvae	Gainesville diet; starvation	Actinomycetota, Pseudomonadota, Bacillota, Euryarchaeota, Bacteroidota	*Actinomyces, Campylobacter, Microbacterium, Enterococcus* and *Enterobacter*
Tanga et al. 2021 [50]	Larvae	Brewers’ spent grains; kitchen food waste; poultry manure; rabbit manure	Bacteroidota, Pseudomonadota and Bacillota	*Dysgonomonas, Parabacteroides, Bacteroides, Flavobacterium, Campylobacter, Lachnoclostridium, Erysipelothrix* and *Enterococcus*
Zhang et al. 2021 [68]	Larvae	Chicken manure	Bacillota and Actinomycetota	Unclassified_f_peptostreptococcaceae, *Enterococcus* and *Turicibacter*
Zhineng et al. 2021 [51]	Larvae	Wheat bran + soybean powder; food waste	Pseudomonadota, Bacillota, Bacteroidota and Actinomycetota	*Enterococcus, Acinetobacter, Providencia, Enterobacter* and *Myroides*
Soomro et al. 2021 [56]	Larvae	Corn flour + bran	Pseudomonadota, Bacillota, Bacteroidota and Actinomycetes	*Morganella, Sedimentibacter, Dysgonomonas, Enterococcus and Providencia*
Cifuentes et al. 2022 [79]	Larvae	Chicken feed	Pseudomonadota and Bacillota	*Stenoytophomonas, Pseudomonas, Alcaligenes, Proteus, Providencia, Morganella, Serratia, Klebsiella, Enterobacter, Brucella, Bacillus, Enterococcus, Mammaliicoccus* and *Lysinibacillus*
Gorrens et al. 2022 [52]	Larvae	Chiken feed; mixture of vegetable coproducts; pig feed; pig feed + wheat bran	Bacillota and Pseudomonadota	*Lactiplantibacillus, Weissella, Enterococcus, Morganella, Providencia, Lactobacillus, Corynebacterium, Proteus, Oceanobacillus, Cerasibacillus, Enterobacter* and *Bacillus*
Pei at al. 2022 [69]	Larvae	Wheat bran; food waste + peanut shell powder	Bacillota	*Bacillus*, unclassified_f_Caloramatoraceae, *Cerasibacillus* and *Gracilibacillus*
Querejeta et al. 2022 [70]	Eggs, larvae, prepupae, pupae, adults	Gainesville diet	Actinomycetota, Bacillota, Pseudomonadota and Bacteroidota	*Acetobacter, Pseudomonas, Delftia, Dysgonomonas, Acinetobacter, Providencia, Myroides, Alcaligenes, Corynebacterium* and *Cutibacteium*
Zhang et al. 2022 [72]	Larvae	Pig manure	Actinomycetota, Pseudomonadota and Bacteroidota	*Enterococcus, Providencia, Dysgonomonas, Koukoulia, Pseudomonas, Sphingobacterium*
Yu et al. 2023 [71]	Larvae	Artificial diet for *Drosophila melanogaster*	Bacteroidota, Campilobacterota, Bacillota, Pseudomonadota, Actinomycetota	*Dysgonomonas, Campylobacter, Enterococcus, Actinomyces, Pseudomonas, Klebsiella* and *Providencia*

**Table 3:** Summary of the taxonomic composition of the mycobiota associated with *H. illucens*

References	Rearing conditions	Substrates	Life stage	Tissue	Methodology	Dominant fungal genera
Cai et al. 2018 [61]	27.5 °C RH: 70%	Wheat bran	Larvae	Whole gut	Illumina HiSeq amplicon sequencing; cultivation followed by partial ITS gene sequencing	*Enlyloma, Lysurus* and *Trichophyton*
Boccazzi et al. 2017 [59]	27 °C RH: 65%	Chicken feed; vegetable waste	Larvae	Whole gut	Pyrosequencing; cultivation followed by partial ITS gene sequencing	*Kazachstania*, *Kluyveromyces*, *Meyerozyma, Alternaria, Davidiella, Meira, Piptocephalis, Botryotina, Collophora, Trichosporon, Rhodotorula, Hypocrea, Eupenicillium, Geotrichum, Pichia, Candida, Debaryomyces*
Zhang et al. 2021 [68]	30 °C RH: Not specified	Chicken manure	Larvae	Whole gut	Illumina MiSeq amplicon sequencing	*Penicillium, Aspergillus, Alternaria, Russula, Phialemoniopsis, Cyberlindnera, Candida, Trichosporon, Trichoderma*
Tanga et al. 2021 [50]	Ambient conditions	Brewers’ spent grain; kitchen food waste; poultry manure; rabbit manure	Larvae	Whole gut	Illumina MiSeq amplicon sequencing	*Pichia, Cyberlindnera, Saccharomycodes, Yamadazyma, Saccharomyces*, and *Scopulariopsis*
Tegtmeier et al. 2021 [44]	27 °C RH: 65%	Chicken feed; cottonseed press cake	Larvae	Whole gut	Illumina MiSeq amplicon sequencing	*Trichosporon, Diutina, Aspergillus, Xeromyces* and *Acaulium*
Klüber et al. 2022 [39]	27 °C RH: 65%	Palm kernel meal	Larvae	Whole gut	Cultivation followed by partial ITS gene sequencing	*Trichosporon, Candida, Sporopachydermia, Lichtheimia, Fusarium, Pichia, Suhomyces, Diutina, Kluyveromyces*
Vitenberg et al. 2022 [75]	Not specified	Household compost	Larvae	Whole gut	Illumina MiSeq amplicon sequencing	*Candidada, Gibberella* and *Meyerozyma*
Yu et al. 2023 [71]	28 °C RH: Not specified	Artificial diet for *Drosophila melanogaster*	Larvae	Whole gut	Illumina MiSeq amplicon sequencing	*Issatchenkia* and *Candida*

In addition to these culture-independent methods, culture-dependent approaches have been used for characterization of the *H. illucens* microbiota, with the successful isolation and culturing of some associated microorganisms, generally followed by their identification by partial 16S rRNA/ITS gene sequencing [45, 49, 54, 58, 59, 61, 65, 66, 72, 73, 79–81]. Culture-dependent approaches have the disadvantage of being laborious and providing only a partial picture of the microbiota associated with an organism, as not all microorganisms are culturable (or are difficult to culture) [82]. However, isolating and culturing a microorganism offers many advantages, including better taxonomic identification, the possibility of acquiring genomic information after genome sequencing and annotation, the opportunity of studying its properties both in vitro and in vivo (e.g., immune challenge with an insect host) and the exploitation of its properties under in vitro conditions with the possibility of using genetic engineering approaches [83, 84]. Thus, while culture-independent approaches provide a quick and comprehensive snapshot of the taxonomic composition of an organism’s microbiota, they remain limited in answering in-depth functional issues, whereas culture-dependent approaches, when integrated into an appropriate experimental workflow (genotyping, phenotyping, use of the strains in subsequent experiments, etc.), can greatly increase the experimental scope for deciphering the function associated with the identified microbial taxa. Some bacterial and fungal associates of the *H. illucens* digestive tract have been cultured and identified, but few studies have yet exploited this valuable reservoir of information through genotyping and phenotyping in particular [80], which are yet prime approaches to unveil the functional dimension of the *H. illucens* gut microbiota. The complementary use of culture-dependent and culture-independent approaches is a paramount strategy for a thorough characterization of the functional diversity of the *H. illucens* microbiota and for its potential exploitation in bioconversion, but it must be supported by complementary approaches.

## The structure of the *H. illucens* bacterial gut microbiota

The insect gut hosts a complex microbial community composed of protists, fungi, bacteria, archaea and bacteria associated with diverse associated effects, ranging from mutualism to parasitism, and whose taxonomic composition can be influenced by various factors [32]. The diversity of the *H. illucens* gut microbiota has been extensively studied in recent years with an emphasis on its bacterial component. Table 1 lists the different studies that have examined the composition of the bacterial microbiota of *H. illucens* and summarizes the rearing conditions, type of substrate used, life stage assayed, tissue assayed, and methodologies used for outlining the microbiota. The diversity of the gut bacterial microbiota of *H. illucens* has been studied primarily from whole larval guts [30, 38, 42–44, 46, 47, 50, 51, 54, 55, 60–62, 64, 66–74, 79, 80] and from whole individuals [40, 48, 49, 52, 53, 56–58, 63, 65]. This is an important limitation because it is now well established that the taxonomic composition of the gut microbiota can differ considerably between compartments of the gut (i.e., foregut, midgut, and hindgut) and even within different parts of a compartment [32]. To date, only two studies have examined the distribution of bacterial communities in distinct parts of the *H. illucens* gut [39, 41]. Another limitation of the studies that have addressed the microbiota of *H. illucens* is that, as discussed above, the taxonomic diversity they report is primarily derived from short-read sequencing approaches, which limits the resolution in identifying microbial taxa and does not allow robust inference of the biological function associated with the identified taxa [77]. Finally, with the exception of a few studies [30, 43, 60, 61, 72, 74], most of these analyses were performed on larvae that had just fed on the contaminated substrate, without a prior fasting period, resulting in biases in establishing the taxonomic composition of the microbiota that is actually associated with the insect gut, since the recorded microbiota likely includes some of that associated with the substrate. Imposing a short fasting period on the larvae prior to molecular analyses is necessary for limiting the detection of substrate-specific microbial taxa and gaining a more accurate picture of the *H. illucens* gut microbiota.

Both independent and culture-dependent approaches have identified more than a dozen bacterial phyla associated with *H. illucens*. Table 2 provides a summary of the taxonomic composition of the bacterial microbiota of *H. illucens* based on the 38 published articles that have addressed this issue. The most dominant bacterial phyla in this insect are Pseudomonadota (Proteobacteria), Actinomycetota (Actinobacteria), Bacillota (Firmicutes), and Bacteroidota (Bacteroidetes), and the most dominant bacterial genera include *Morganella*, *Providencia*, *Dysgonomonas*, *Ignatzschineria*, *Enterobacter*, *Proteus*, *Enterococcus*, *Bacillus*, *Klebsiella*, *Citrobacter*, *Scrofimicrobium* and *Actinomyces*. Most studies that have addressed the *H. illucens* microbiota have focused on the factors that may shape its composition, primarily the substrate, but also the host developmental stage, the rearing temperature, and the genetic variability of the host and the bacterial strains. Overall, these studies show that the *H. illucens* gut microbiota is highly diverse despite the presence of some dominant bacterial taxa and is highly dynamic, shaped by a range of factors that are discussed below.

### The influence of the food substrate

Most studies that have examined the factors shaping the bacterial gut microbiota of *H. illucens* have focused on the impact of the rearing substrate on the diversity of bacterial communities hosted by the larvae [30, 38–52, 54, 55]. Indeed, the diet is the primary source of microbial diversity associated with the animal digestive tract, but it also acts as a selective constraint favoring the establishment in the digestive tract of microorganisms that meet specific host requirements for nutritional services (e.g., via the multiple catalytic properties of bacteria required for the degradation of various organic compounds) [32, 85]. These studies pinpointed two major trends in the interaction between the substrate and the *H. illucens* gut microbiota. The first trend is that the taxonomic composition of the microbial communities associated with the larval gut and the relative abundance of the microbial taxa depend on the nature of the food substrate [38, 39, 41, 42, 44–50, 55]. However, this trend is supported by somewhat mixed findings. For instance, Zhan et al. 2019 [41] report that larvae fed dairy and swine manure tend to have a more diverse microbiota than those fed poultry manure, suggesting that the diversity of the gut microbiota largely reflects the diversity of bacteria that thrive in the substrate and succeed in infecting the larvae. However, Klammsteiner et al. [42, 47] temper the role of substrate in structuring the gut microbial communities of *H. illucens* by showing that larvae feeding on very different types of substrates exhibit a similar gut microbiota in terms of taxonomic diversity with the systematic presence of some dominant taxa whose relative abundance depends on substrate. Tanga et al. 2021 [50] report an in-between trend, finding significant differences in the taxonomic composition of the gut microbiota as a function of substrate, but with the systematic presence of six bacterial genera - *Dysgonomonas*, *Morganella*, *Enterococcus*, *Pseudomonas*, *Actinomyces* and *Providencia* - differing in relative abundance depending on substrate. The authors suggest that the taxonomic differences in the composition of the gut microbiota are the result of the addition of conserved genera and substrate-specific transitive bacterial associates. These studies were performed with different experimental settings, which may explain the different trends reported. However, the regular occurrence of specific bacterial genera in the *H. illucens* gut microbiota and the multitude of more sporadically associated genera reported in other studies suggests that the bacterial microbiota of *H. illucens* is actually composed of substrate-specific associates and a few well-preserved genera, with the relative abundance of each taxa varying with the substrate. While all these studies show that the nature of the substrate is a major factor in the structuring of the *H. illucens* gut microbiota, the services that the different bacterial associates could specifically provide to the insect during bioconversion remain elusive.

A second important trend is the modification of the gut microbial community structure during bioconversion. While some studies report dynamic variation in the composition of bacterial taxa as well as their relative abundance [30, 62], others report a dynamic variation primarily in the relative abundance of the dominant taxa [43]. This trend can be explained by the fact that the structure of microbial communities thriving in the substrate tends to change dynamically as a function of its transformation, resulting in a modification of that associated with the insect’s digestive tract [30, 62, 68]. In studying the bacterial communities structure of *H. illucens* during consecutive industrial rearing cycles, Gorrens et al. 2022 [52] found the bacterial composition of the larvae and substrate shift over two time points within one rearing cycle, confirming that the larval intestinal bacterial community structure is dynamic during bioconversion. These observations also tend to confirm that the larvae modify the bacterial community structure of the substrate [30, 68, 86] and therefore that substrate and larva shapes each other’s bacterial community composition during bioconversion. However, the authors extended their observation to several rearing cycles and found fairly low inter-cycle variability in the larval bacterial community during consecutive rearing cycles, suggesting the existence of a core bacterial microbiota associated with *H. illucens*.

Finally, while some trends could be identified regarding the influence of the substrate on the structure of the *H. illucens* microbiota, it should be kept in mind that some abiotic and biotic factors may interfere with the interplay between the substrate and the larval microbiota (e.g., host genotype, rearing temperature conditions, experimental design, etc.) [52]. This may explain why some studies show that the taxonomic composition of the larval microbiota reflects that of the substrate, while others report more mixed results [40, 42, 47, 48, 54]. This lack of a clear trend could also be explained by the continuous reorganization of the substrate microbiota structure during bioconversion, with a taxonomic composition that does not match that of the gut microbiota at the same time. In addition, larval rearing is done in an open environment that can promote the continuous flow of environmental microbes that can contaminate the substrate and the larvae. Despite all these caveats, it is now evident that the substrate contributes significantly to the establishment and structuring of the larval microbiota [39, 65]. Larvae do not grow well on a sterile substrate, suggesting that it is from a contaminated substrate that they acquire the microorganisms that form their microbiota [87]. This also highlights the importance of the gut microbiota for insect health, nutrient recycling and digestion. *H. illucens* larvae lack the necessary enzymes to efficiently digest complex compounds such as cellulose and lignin and its gut microbiota is capable of producing a range of enzymes, including cellulases, which are essential for breaking down complex molecules present in ingested organic matter [38, 80]. At the same time, bioconversion by larvae dynamically shapes the substrate microbiota which results in a decrease in substrate microbial diversity during the process [30, 68]. The question now is whether the microbial partners that *H. illucens* acquires from its substrate are stably maintained solely by transfer from the environment to the insect (i.e., horizontal transmission) or whether some bacterial associates take the route of vertical transmission that allows them to pass from one generation to the next.

### The influence of developmental stage

The bacterial community structure of *H. illucens* at different life stages has been examined in a few studies, all of which revealed that bacterial taxonomic diversity and the relative abundance of taxa shared between different developmental stages varies with insect development [47, 57, 62, 70]. Only Zheng et al. 2013 [57] and Querejeta et al. 2022 [70] investigated microbiota change over the entire life cycle of *H. illucens*, i.e., from egg to adult stage. Both studies report that some bacterial genera are commonly found in all developmental stages (e.g. *Providencia* and *Alcaligenes*), suggesting the existence of a core microbiota. On the other hand, some genera are shared only between larval stages (e.g. *Morganella* and *Enterococcus*) and are absent from eggs and adults, suggesting that the composition of bacterial communities associated with *H. illucens* is modulated according to the bacterial services specifically required at each developmental stage of the insect’s life cycle [70]. Cifuentes et al. 2020 [62] and Klammsteiner et al. 2021 [47] looked at the structure of bacterial communities as a function of different larval stages and report the same trend: a fairly stable gut microbiota whose composition does not change significantly during larval development when insects are reared on the same substrate. Although these studies provide insight into the dynamics of microbiota composition as a function of insect development, further studies are needed to understand how changes in bacterial communities occur during insect development, particularly from eggs to larval stages and from larval stages to adults.

### The influence of genetic variability

Genetic variability is another factor that can influence the composition of the *H. illucens* gut microbiota. Indeed, genotype-by-genotype interactions condition the coevolution between the host and its microbial partners, and the outcome of infection is largely the result of the genotypic diversity of both parties (e.g., at the immune level for the host and at the level of colonization factors for the microorganism) [88, 89]. So far, regarding *H. illucens* and its microbiota, it is more the genetics of the host that has been emphasized than that of its microbiota. Khamis et al. 2020 [63] analyzed the microbiota composition of *H. illucens* individuals collected from diverse regions around the world and found that the bacterial communities differed greatly between geographical origins, probably due to different diets combined with the genetic diversity of the insects sampled. Using different *H. illucens* strains fed on different substrates, Greenwood et al. 2021 [46] demonstrated that the gut microbiota is shaped by both environmental factors (the substrate in their study) and host genetics. How partner genetics shape the *H. illucens* microbiota is an important issue that remains neglected in most current studies and should be further investigated, especially to better determine the nature of interactions between the host and the microorganisms it harbors and the genetic factors that condition the establishment of these associations (e.g., immune defenses, virulence factors, etc.). The genome of *H. illucens* was recently sequenced from two insect lineages, providing the foundation for such studies [41, 90]. Nevertheless, genome sequencing of a larger number of *H. illucens* lineages could provide valuable information on the intraspecific genetic variation of the insect and its influence on the microbiota. Regarding the microbiota of *H. illucens*, although many bacterial strains have been isolated and identified, the investment in sequencing and annotation of their genome remains very limited at this time. Yet, these genomic data are essential for a thorough taxonomic characterization of the isolated strains, but also to better identify their potential biological function and the nature of the interactions that these bacteria have with *H. illucens*. The use of metagenomic approaches is another alternative to capture the genomes of bacterial associates present in the gut [91]. One limitation of these approaches is the high proportion of insect DNA, which prevents efficient sequencing of the bacterial DNA present in much lower proportions. However, fractionation methods can be used to enrich DNA extractions with bacterial DNA to improve sequencing yield [92]. In the near future, increased emphasis on these genomic aspects would allow the identification of functions associated with these microbes and possibly initiate the exploitation of their properties outside the host [93].

### The influence of temperature

It is well known that thermal conditions can strongly affect the composition of the microbiota associated with a multitude of eukaryotic species [94, 95]. To date, only Raimondi et al. 2020 [65] have addressed the influence of rearing temperature on the composition of the microbiota of *H. illucens*. In particular, they found that at the prepupal stage, increasing rearing temperature was associated with a decrease in the relative abundance of *Providencia*, one of the genera most frequently associated with *H. illucens*, and an increase in the relative abundance of other genera, including *Bacillus*, *Proteus*, *Bordetella* and *Alcaligenes*. However, they did not observe a significant effect of temperature on the taxonomic composition of the microbiota. Examining how temperature affects the microbiota of *H. illucens* is essential, as it could influence the propensity of certain beneficial microorganisms to multiply and perform their services, thus improving host fitness and, consequently, could have a significant impact on bioconversion efficiency [27]. In addition, temperature could influence the way the microbiota of the substrate is structured [96, 97], which in turn could influence the structuring of the *H. illucens* gut microbiota. Finally, temperature could also condition the propensity of some pathogens to multiply in the substrate and in the larvae, which could affect the health of the reared insects, but more importantly, human or animal health in the consumption cycle [65].

### The influence of toxic compounds

A few studies have addressed the effects of toxic compounds in organic wastes on the microbiota of *H. illucens*, including antibiotics that are particularly abundant in manures. They indicate that antibiotics can influence the structure of the *H. illucens* microbiota, which in turn contributes to the detoxification of these compounds. Larvae feeding on an oxytetracycline-enriched diet are able to degrade antibiotics, presumably through antibiotic-resistant bacteria whose relative abundance tends to increase [64, 67]. Similar results have been obtained for tetracycline [61], paving the way for biodegradation of pharmaceuticals by insect bioconversion. Antibiotic resistance genes (ARGs) in animal manure are an environmental problem, as natural bacteria are exposed to this waste and develop multi-drug resistance, which can lead to bacterial resistance outbreaks [98]. *H. illucens* larvae and their associated gut microbiota contribute to ARGs degradation during the digestion process, which is accompanied by changes in bacterial community structure with reduced representation of potentially pathogenic bacterial taxa [60]. One hypothesis is that *H. illucens* larvae, through activation of their immune system, can both reduce the amount of ARGs and limit the growth of ARG-carrying bacteria, which could have beneficial health consequences since the emergence of multidrug-resistant bacteria is a major public health problem. Finally, heavy metals such as cadmium and copper, often present in high concentrations in animal manure, tend to alter the taxonomic composition of the gut microbiota of *H. illucens*, but without affecting the development and health of the insect [74]. All these observations indicate that the function of the gut microbiota is not limited to digestion per se and could fulfill a detoxification role, paving the way for the use of *H. illucens* to remediate organic waste from hazardous wastes that may pose a risk to ecosystems and public health.

### The influence of the experimental setting

The diversity of the experiments conducted so far has allowed to highlight the influence of some specific abiotic and biotic factors on the composition of the microbiota and to evidence the existence of dominant bacterial taxa. However, the variety of experimental parameters used also limits comparative analyses between studies and may overshadow some general trends regarding how the *H. illucens* gut microbiota is structured. For example, the different studies sometimes use very different temperature and humidity regimes (Table 2). The substrates used in standard rearing are also generally very different and their precise composition is unknown because they are based on the use of raw materials. *H. illucens* is an emerging model insect whose functional diversity of the microbiota is mainly studied in a bioconversion context with the use of a variety of organic wastes used as substrate. However, establishing basic standard rearing conditions (including the use of a standard diet with a known and reproducible formula or standard temperature conditions) would have the benefit of minimizing confounding factors, untangling contrasting observations, and making comparative analyses between studies more relevant [99], ultimately allowing access to more robust conclusions about how the gut microbiota of this insect is shaped.

## Does *H. illucens* harbor a core (bacterial) microbiota?

Defining the core microbiota of an insect species is essential in the study of the functional diversity of insect microbiota, as shared microbial taxa are considered the most ecologically and functionally important microbial associates of that host [100]. It is generally defined as the microbial taxa shared by two or more microbial communities in a given host species or environment [101]. Several studies suggest that, like many insect species, *H. illucens* harbor a core bacterial microbiota capable of persisting in the host through successive life stages [42, 52, 53, 57, 79]. However, the taxonomic composition of the core microbiota can vary greatly from one study to another depending on the experimental design, the substrate used and a range of other factors. Recently, IJdema et al. 2022 [35] re-analyzed 16 S rRNA gene sequence data sets from 11 surveys to test the hypothesis that *H. illucens* harbor a core microbiota. They found that members of the Enterococcus and *Morganella* genera were present in over 80% of the samples. The genus *Providencia* was found in over 75% of the samples. The genera *Scrofimicrobium* and *Klebsiella* were also frequently found associated with the *H. illucens* digestive tract. However, although these genera are frequently associated with *H. illucens*, their prevalence is not 100%, suggesting that this insect species does not harbor a core microbiota in the strict sense, as is the case for insect taxa that have evolved obligate relationships with heritable symbionts for nutrient acquisition and that are fixed bacterial partners in host species [102]. Furthermore, there is currently no evidence that some associates of *H. illucens* are capable of undergoing vertical transmission, a process that is indicative of a stabilized mutualistic relationship that can be inherited from generation to generation [103]. The bacteria forming the core-microbiota of the *H. illucens* gut, if they render services to their host, are thus facultative partners that probably derive from the microbial flora of the substrate. For the moment, the functions specifically associated with these bacterial associates remain elusive. The bacterial diversity inhabiting the *H. illucens* gut may include associates providing beneficial services to the host in a mutualistic relationship. However, it may also include members with a strong propensity to multiply in decaying material and within the insect itself because they possess the arsenal to bypass the host’s immune defenses and colonize the digestive tract opportunistically as commensals or pathogens [32].

The genera *Enterococcus*, *Morganella*, *Providencia*, *Scrofimicrobium*, and *Klebsiella* are those most frequently detected in *H. illucens* [35]. These are therefore the genera that should be examined as a priority to reveal their associated effects and potential functions in *H. illucens*. To do so, culture-dependent approaches are needed to isolate species and strains of these target candidates to (1) sequence and annotate their genomes (e.g. to analyze their repertoire of virulence factors, metabolic capacity, etc.), (2) characterize their properties in an in vitro setting (in particular their ability to degrade specific organic compounds, express virulence factors and produce antimicrobial compounds), and (3) examine their effects on insects to determine whether they are commensal, mutualistic, or pathogenic associates (e.g., through immune challenge experiments). Most metabarcoding studies performed on *H. illucens* have used short-read sequencing approaches that eventually allow identification down to the genus, but rarely down to the species. In addition, a bacterial species may itself comprise a myriad of strains and phylotypes with extremely diverse phenotypes and associated effects. Thus, in addition to culture-dependent approaches, it is also now timely to use more specific approaches, including long-read metabarcoding, multilocus sequence typing and shotgun metagenomics to surface a picture of the *H. illucens* gut microbiota that better considers the inter-strain diversity [104–106].

Finally, an issue that has not been examined to date, despite the detection of egg-associated bacteria [57, 58, 70, 107], is the possibility that members of the gut microbiota experience vertical transmission, i.e., transmission from parents to offspring. This is an important issue because this transmission route ensures generational continuity of infection and associated benefits and is a sign that the antagonistic aspects of the host-symbiont interaction are fading and the relationship is stabilizing through the evolution of an interdependent relationship [108]. Although vertical transmission usually occurs internally by transovarial transmission, it can also occur by external mechanisms, such as the placement of symbionts (“egg smearing”) or capsules containing the symbiont on eggs by the female [109]. The hypothesis of vertical transmission of gut symbionts in *H. illucens* still needs to be tested to clarify the degree of interaction they have evolved with their host.

## Spatial distribution of the bacterial microbiota in the digestive tract

The digestive tract of insects is sometimes wrongly perceived as a single organ and molecular analyses are often performed on the whole system. Almost all studies that have examined the gut microbiota of *H. illucens* have, in fact, been performed on the entire digestive system, without regard to its anatomical and functional complexity (Tables 1 and 2). However, the digestive tract of insects, similarly to that of vertebrate animals, is subdivided into several anatomical regions: the foregut, midgut and hindgut [32]. The midgut is generally the primary site of digestion and nutrient absorption, while the hindgut is the site of water reabsorption and absorption of some nutrients. The basic design of the gut is similar among all insects but exhibits anatomical modifications that reflect adaptations to specialized niches and feeding habits, and result in compartmentalization of gut microbes into specific parts of the gut. Deciphering the spatial distribution of microbial communities in different parts of the gut is therefore essential to identify the functions with which they are associated.

**Fig. 1:**
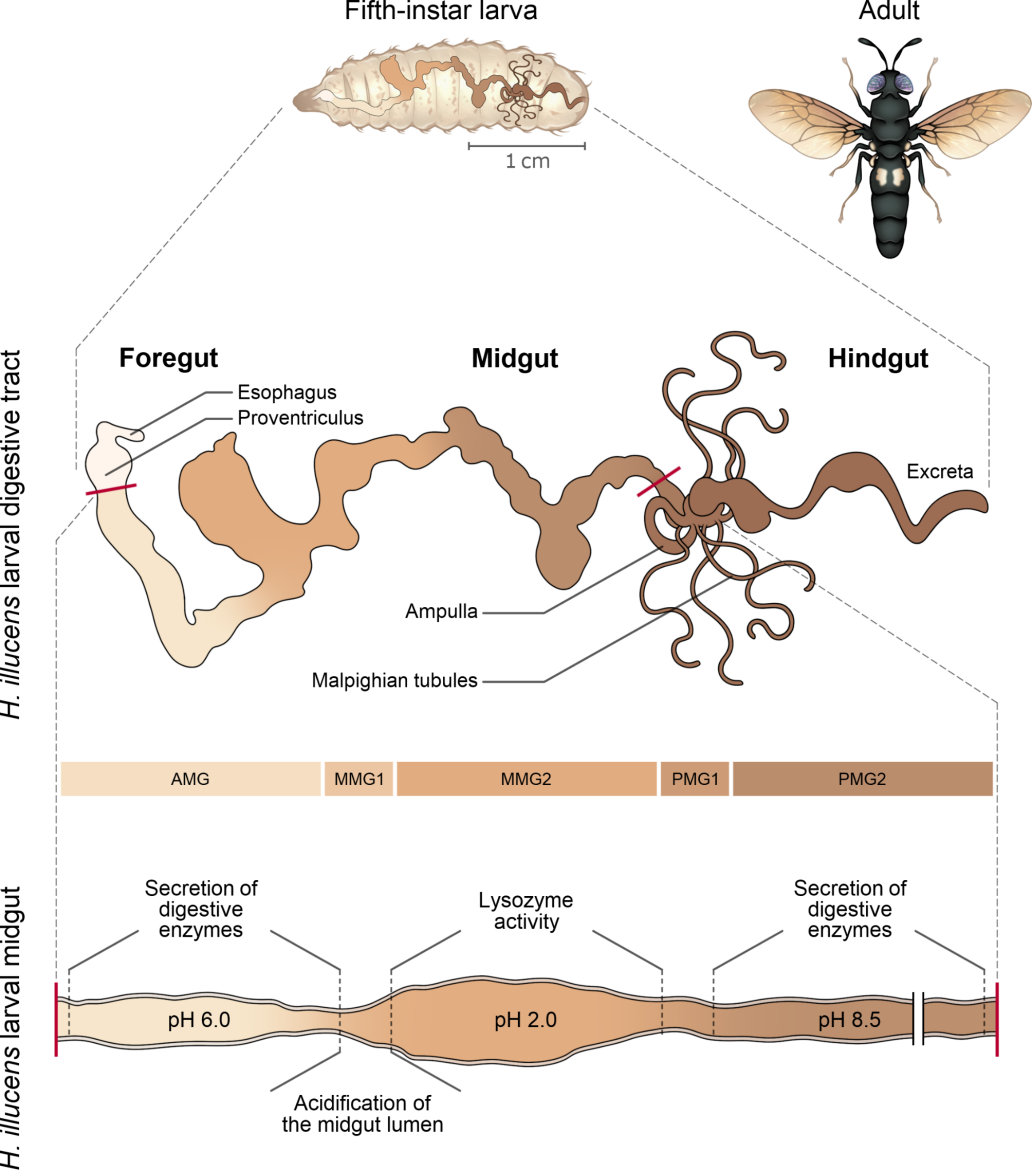
Schematic representation of *H. illucens* larval digestive tract. The digestive tract of this insect species is characterized by an extremely long midgut. This is divided into distinct regions characterized by specific physico-(bio)chemical conditions and digestive activities. Bonelli et al. 2019 [110] showed that each region of the midgut harbors distinct bacterial communities, suggesting a specific compartmentalization of microbial partners according to digestive processes. AMG, Anterior midgut; MMG, Middle midgut; PMG, Posterior midgut.

The anatomy of the digestive tract of *H. illucens* has only recently been described [39, 110–112]. Its most striking anatomical feature is that the midgut is remarkably long and subdivided into several regions (Fig. 1). Unfortunately, most metabarcoding studies have focused on depicting the bacterial microbiota at the level of the whole gut, i.e., without fractionation of the digestive tract prior to DNA extraction (Table 1), which does not provide any information regarding how the bacterial communities are spatially distributed in the system. Yet, this information is essential to appreciate the possible functions, for example in digestion, associated with the bacteria that compose the microbiota. Bruno et al. 2019 [39] is the only metabarcoding study that considered the spatial distribution of bacterial communities in the digestive tract but focused only on the midgut and its different anatomical regions. The authors found that different parts of the midgut (anterior, middle, posterior) are featured by different physico-chemical conditions and harbor bacterial communities that differ in terms of taxonomic composition and density. Regardless of the substrate on which the larvae fed, they found that the anterior part of the midgut is characterized by high microbial diversity, which progressively decreases from the anterior to the posterior part, and that each part is characterized by a different bacterial load, which is higher in the posterior midgut than in the anterior midgut. To explain these results, the authors hypothesize that specific mechanisms in the middle midgut would select the entry of specific bacterial taxa into the posterior midgut. By showing that the overall gut microbiota does not reflect the composition of the microbiota of each part of the midgut, the authors demonstrate the relevance of working on the distinct anatomical regions of *the H. illucens* digestive tract. The authors do not comment exhaustively on the function of these different parts of the midgut in digestion, nor on the function that the bacterial communities might perform in their respective compartments. These are important aspects to consider in future studies to unveil the functional dimension of the diversity of bacteria that compose the *H. illucens* gut microbiota. For this, a more in-depth understanding of the biology of the digestive system of this insect is needed. Microscopic approaches can provide a complete picture of the complexity of its anatomy [110, 112], but could also be used to reveal the specific localization of bacterial associates in the different regions of the digestive tract [113]. It has recently been reported that the presence of a gut microbiota triggers significant changes in the transcriptional profile of *H. illucens* during the larval ontology, suggesting that the interaction induce intense regulation of host functional genes [114]. Gut-focused transcriptomic approaches could reveal mechanisms (e.g., specific immune responses) that are differentially activated in the different compartments to regulate the gut microbiota and shape its spatial distribution [115].

## Functions associated with the gut microbiota of *H. illucens*

The study of the *H. illucens* microbiota is recent and its functional diversity has only recently been examined. The functions and effects associated with bacteria residing in the *H. illucens* gut currently rely largely on functional inference based on short-read metabarcoding data and are therefore highly speculative. For example, several studies have used the PICRUSt software [116] to sketch potential functions associated with the bacterial microbiota of *H. illucens* [67, 68, 71]. Inferring microbiota functions from taxonomic genes is a fast and inexpensive approach, but it lacks robustness because it relies only on the use of part of a marker gene and is highly dependent on available reference genomes. Furthermore, it takes little account of the intra-species diversity of bacteria while strains belonging to the same species may be associated with very different induced phenotypic effects [77].

In the absence of hard evidence regarding the functions associated with members of the *H. illucens* microbiota, we limit ourselves in this review to outline the putative biological functions of some of the most frequently identified bacterial genera in *H. illucens*, including *Enterococcus*, *Morganella*, *Providencia*, *Klebsiella*, *Dysgonomonas* and *Lactobacillus* [35]. Members of the *Enterococcus* genus can be associated with various effects: they can be pathogenic, assist the host by providing immune-related antimicrobial peptides, and participate in the degradation of plant polymers through their complex carbohydrate-degrading enzymes and nitrogen, hydrogen and sulfur metabolism [117–120]. A culture-dependent approach has shown that *H. illucens* may in fact harbor several *Enterococcus* phylotypes and therefore members of the genus potentially associated with different effects [79]. Members of the genus *Morganella* may also be pathogens, while some strains may play a role in urea hydrolysis or produce phenol which serves as a sex pheromone in some insect species [121–123]. *Providencia* bacteria may play an important role in protein and lipid conversion in the gut, in degradation of antibiotics, and has been proposed to play a role in protein and lipid conversion in the *H. illucens* gut and to produce xylanases, which may help the insect digest hemicellulose from plant cell walls [43, 64, 124]. The genera *Dysgonomonas* and *Lactobacillus* are also frequently detected in *H. illucens* larvae. Members of *Dysgonomonas*, known for their ability to degrade complex polysaccharides, could contribute to lignocellulose degradation in the digestive tract of *H. illucens* [125]. *Lactobacillus* members could have protective effects by detoxifying pesticides and xenobiotics and increasing the expression of antimicrobial peptides limiting the establishment of pathogenic bacteria [126, 127]. The effects potentially associated with the most dominant bacterial members of the *H. illucens* microbiota reported here represent only a fraction of the hypothetical roles reported in the existing literature are primarily based on inferences from taxonomic genes and are therefore, at this time, fairly speculative. Genomic and experimental data allowing for better profiling of the identified bacteria are needed to grasp the functions of the *H. illucens* microbiota. The isolation and culture of bacteria residing in the gut of *H. illucens* is a first step in this effort [45, 49, 54, 58, 61, 65, 66, 72, 73, 79, 80]. Callegari et al. 2020 [80] examined the hydrolytic capabilities of a collection of bacteria isolated from the digestive tract of *H. illucens* and demonstrated their ability to degrade a variety of organic compounds in the rearing substrate and found that *Bacillus licheniformis* HI169 and *Stenotrophomonas maltophilia* HI121 isolates were able to enhance the growth of *H. illucens* when supplemented in the substrate. These results are a first step in experimental validation of the contribution of the microorganisms to the *H. illucens* growth and development. Antimicrobial properties of the *H. illucens* gut microbiota against foodborne pathogens have also been demonstrated experimentally [66]. The deciphering of the functions associated with the *H. illucens* microbiota has only just begun and should soon open up prospects for industrial applications. Furthermore, the combined use of metagenomic and (meta)transcriptomic approaches, which remain poorly performed in the study of the digestive tract of this insect, is crucial to determine the functions of the *H. illucens* gut microbiota [128–130].

## Beyond the bacterial dimension: the mycobiota and the virobiota

While the study of the taxonomic composition of the *H. illucens* microbiota has primarily focused on bacterial communities, some studies have examined other components of the microbiota, including fungi (the mycobiota) and viruses (the virobiota). To date, seven studies have examined the mycobiota of *H. illucens* gut, either using culture-dependent or metabarcoding approaches using the internal transcriptional spacer (ITS), and sometimes using a combination of both approaches [44, 50, 59, 61, 68, 71, 81]. Table 3 lists the different studies that have addressed the taxonomic composition of the mycobiota of *H. illucens*. These studies reveal that *H. illucens* can host a wide variety of fungi, mainly belonging to the phylum Ascomycota and including, among others, the genera *Pichia*, *Candida*, *Diutina*, *Kluyveromyces*, *Trichosporon* and *Fusarium*. Several of these studies have shown that the fungal microbiota profiles in *H. illucens* is largely influenced by the nature of the substrate [50, 59, 66, 75]. For example, plant waste tends to be associated with a greater diversity of fungal species compared to chicken feed [59]. These studies also suggest that *H. illucens* does not harbor a core-mycobiota, although some fungal genera are frequently associated with the species, particularly *Pichia* and *Candida* [50, 59, 68, 71, 81]. Interestingly, Zhang et al. 2021 [68] demonstrated that *H. illucens* larvae change the microbial communities present in the substrate and harbor a mycobiota that does not mirror that of the substrate, suggesting that the insect is not a passive recipient of the fungal flora evolving in its diet and that, as is the case with the bacterial component of the microbiota, specific interactions can occur between the insect and fungal associates.

As for the bacterial microbiota, the nature of the interactions between *H. illucens* and its mycobiota remains largely unknown and is based on assumptions. It is hypothesized that the fungal community inhabiting the gut may play an important role in detoxifying metabolites and providing enzymes, essential amino acids, vitamins, and sterols in the host diet [131]. *Pichia* yeasts and some *Fusarium* species, are well known to digest cellulose and thus could enhance its digestibility in host larvae [81]. On the other hand, the genus *Fusarium* also includes pathogenic species and strains. For example, Klüber et al. 2022 [81] isolated and identified a strain of *Fusarium solani* with highly pathogenic effects on *H. illucens* and that could represent a risk for livestock production. Some fungi, such as those of the genus *Trichosporon*, may have antibacterial activity that contributes to the elimination of pathogenic bacteria, including foodborne pathogens that pose a risk to human and animal health [132, 133]. Interestingly, none of the studies on the *H. illucens* mycobiota reported strains that could release mycotoxins that may pose a risk to animals and humans when they enter the food web. However, the study of the mycobiota of *H. illucens* is still in its infancy and needs to be refined to better understand the functions it performs in the insect’s digestive tract, but also to define rearing conditions that limit the growth of pathogenic fungi and/or the expression of mycotoxins (usually expressed at elevated temperatures) that may be detrimental to the health of the insect and possibly to other links in the food chain [134].

The viral component of the *H. illucens* microbiota, the virobiota, has been even less examined since only one study has specifically addressed it [135]. The authors performed an *in-silico* analysis consisting of a screening of the *H. illucens* transcriptome available in public databases. Their analysis suggests that *H. illucens* is the recurrent host of *Totiviridae*, a family of double-stranded RNA viruses. Nevertheless, the exact nature of these interactions remains unknown. *H. illucens* larvae have been shown to effectively reduce the viral load in substrate contaminated with different types of viruses (Orthoreovirus, Mastadenovirus, Teschovirus) [136]. Yet, although *H. illucens* likely interacts with a variety of viruses in its environment, no virus typically associated with this insect has yet been detected in vivo. Mass-reared insects typically deal with a range of viruses that need to be known and detected to prevent production losses or even prevent any risk of transfer of viral pathogens from the insects to other links in the food chain [137]. Unlike bacterial diversity, viral diversity cannot be monitored by metabarcoding approaches. However, metagenomic approaches can provide insight into the composition and structure of viral communities inhabiting insects [138] and their use will be essential in lifting the veil on the interaction between *H. illucens* and the virosphere.

## The pathogenic dimension of the *H. illucens* microbiota

Most of the ontogenic development of *H. illucens* takes place in decaying organic matter which is an environment rich in microorganisms of all kinds that can be competitors for resources but also potential pathogens for which the insect can be a dispersal vector. In this context, examining the pathogenic nature of the gut microbiota of *H. illucens* is fundamental in a mass rearing context for food and feed production for two main reasons. First, for the health of the livestock and its production, on which profit depends. Indeed, mass rearing of insects can cause epidemics in the production system that can rapidly lead to decimation of livestock [139]. Second, for sanitary reasons. It is indeed important to identify potential foodborne pathogens that could emerge from the use of *H. illucens* as feed and food. A few review articles have already addressed the issue of biological contaminants in mass rearing of insects, focusing on insect immunity, rearing conditions that prevent pathogen multiplication and food safety legislation [34, 140–142]. In this paper, we focus on the diversity of the entomopathogenic component of the *H. illucens* microbiota and the microbes that may pose a health threat to downstream links in the food chain and suggest research avenues to study these microorganisms and their interaction with the host.

Like any mass rearing, insect farming can be challenged by diseases that can rapidly spread and decimate insect populations [143]. In their review articles, Joosten et al. 2020 [140] and Barrett et al. 2023 [142] provide a list of infectious agents (fungi, viruses, bacteria, and protozoa) that have been described as entomopathogenic in Diptera and are suspected to be a potential threat to *H. illucens* farms. However, to date, no significant outbreaks caused by a pathogen have been reported on any *H. illucens* farm, or even symptoms of disease, and the pathogens that could cause disease in this insect are unknown. However, two recent studies report the existence of two pathogens that can increase larval mortality. Klüber et al. 2022 [81] reported the pathogenic effect of the filamentous fungus *F. solani*. However, these experiments were performed by injecting the pathogen directly into the hemolymph, and there is no evidence that this fungus can overcome the defensive barriers of the digestive tract, the primary route of entry for an infection. She et al. 2023 [144] identified *Poenibacillus thiaminolyticus* as a pathogen of *H. illucens* larvae whose virulence increases with high rearing temperatures. The lack of disease observed in *H. illucens* may be explained by the fact that mass rearing of this insect species is quite recent and therefore few pathogens could be observed compared to insects that have been mass reared for many years. However, because *H. illucens* is adapted to feed on a wide range of substrates teeming with a variety of microorganisms, another hypothesis is that this insect species is endowed with a robust immune system that allows it to cope with pathogenic microbes that are harmful to other insect species [145]. However, very little is known about the immune system of *H. illucens* and it is the *Drosophila melanogaster* immune system that is often used to speculate on the immune capabilities of *H. illucens* [140]. The recent sequencing of the *H. illucens* genome should enhance our understanding of the insect’s immunity [41, 90], especially the chromosome-level genome assembly provided by Generalovi et al. 2021 [90]. Indeed, this high-quality assembly will allow annotation of the immune and stress gene repertoire to obtain a comprehensive picture of the immune and defense mechanisms that *H. illucens* may use to cope with microbes. The thorough annotation of this gene repertoire is also crucial for conducting robust immune challenge experiments coupled with transcriptomic analyses to identify the defense mechanisms that are actually activated in the different compartments of the gut and that control the populations of resident microorganisms, whether they are pathogenic or engaged in mutualistic interactions with the insect. Finally, annotation of the immune and stress gene repertoire will shed light on the diversity of antimicrobial peptides (AMPs) that *H. illucens* is able to express. Indeed, several experimental studies suggest that *H. illucens* can express and release a wide variety of AMPs that would allow this species to cope with the diversity of microbial agents it encounters in its food substrate or in the environment [130, 146–152].

Organic waste is a breeding ground for various microbes, some of which are pathogenic to animals and humans. When larvae that feed on these wastes are processed into food or feed products, the presence of biohazardous microbes must be monitored during rearing and processing to ensure a safe final product [153]. Numerous studies on the microbiota of *H. illucens* have examined whether this insect is capable of accumulating pathogens that may pose a health risk to animals and humans. One major finding is that *H. illucens* drastically alters the microbial diversity of the substrate it feeds on and tends to reduce the load of pathogenic bacteria including *Salmonella* spp., *Enterococcus* spp., *Escherichia coli* and *S. aureus* that are initially present in organic waste [68, 72, 133, 136, 154–158]. Zhang et al. 2021 [68] showed that this sanitizing effect of *H. illucens* also applies to fungi growing in the substrate and Lalander et al. 2014 [136] to viruses. This antimicrobial activity could come from the insect itself [127, 159], but also from its gut microbiota [129–131] as suggested by Gorrens et al. 2021 [133] and Zhang et al. 2022 [72]. The Gorrens et al. 2021 study shows that *H. illucens* virtually eradicates *S. aureus* from the substrate, but also that the pathogen load in the larvae is very low and that *Trichosporon*, a frequent fungal associate of the *H. illucens* gut, exerts strong antimicrobial activity against *S. aureus*. Zhang et al. 2022 showed that the presence of pathogens influences the composition of the *H. illucens* gut microbiota and that *Bacillus* strains isolated from *H. illucens* larvae exerted strong antimicrobial activity against *S. aureus*. These two studies suggest that the *H. illucens* gut microbiota may play a primary role in eradicating pathogens from both the substrate and the *H. illucens* gut. However, it seems that these results cannot be generalized to all foodborne pathogens and further studies are required to clarify the sanitizing role of the *H. illucens* microbiota [160]. Nevertheless, these studies pave the way for the use of members of the *H. illucens* microbiota for the control of foodborne pathogens and for the discovery of AMPs that could be used for various applications [161].

Furthermore, despite the sanitary properties of *H. illucens* on the substrate, it appears that some foodborne pathogens can be found in the rearing residues [40] and in the larvae, such as *Campylobacter spp*, *Clostridium spp*, and *B. cereus* [30, 162]. In addition, 16 S rRNA gene profiling reveals the abundance in larvae of certain genera (e.g., *Myroides*, *Proteus*, *Providencia*, and *Morganella*) that may be beneficial associates for *H. illucens* but putative opportunistic pathogenic strains that may pose a health risk to animals and humans [163–168]. The sanitary aspects are crucial in the context of the development of mass rearing of insects potentially dedicated to feed and food and the pathogenicity of the microbes growing in the *H. illucens* larvae must be carefully examined. To date, the study of *the H. illucens* microbiota has relied mainly on culture-independent approaches that provide very little information on the phenotype that the identified microorganisms may express (including pathogenicity). The use of more specific detection methods, genomic analyses and phenotyping approaches performed on the collection of microorganisms isolated by culture-dependent methods is a pivotal step to clarify the pathogenic dimension of the *H. illucens* microbiota [169]. This step is essential to ensure sound monitoring practices to avoid the introduction of foodborne pathogens into the animal and human food chain [34, 142]. It is also essential to formulate good hygienic practices and adequate sanitary guidelines for microbiological optimization of *H. illucens* rearing conditions and post-harvest treatments of larval-derived end products dedicated to consumption (e.g., by heat pasteurization, freeze-drying, hot air-drying, UV treatment, acidification or any other practice that can reduce the load of pathogens) [170, 171].

## Conclusions

The black soldier fly *H. illucens* has recently emerged as one of the most important insects for bioconversion and is now being reared by multiple companies on an industrial scale. This craze is due to the fact that the larvae can feed on a multitude of organic materials to rapidly generate valuable biomass for multiple purposes. In recent years, many studies have focused on the functional diversity of the *H. illucens* microbiota in relation to its diet, as reviewed in this paper. This microbiological component is increasingly considered to optimize insect biomass production in a biosafety context, but also because it could be leveraged for the development of new biotechnological tools [33].

## Perspectives and future directions

Beyond these applied aspects, *H. illucens*, with its exceptional digestive capacities and complex gut microbiota, constitutes a fascinating model to address important fundamental questions about the interactions that insects have evolved with microorganisms. For example, what specific adaptations does this insect endow to cope with such a diversity of microbial associates? How are these microorganisms distributed and regulated in different parts of the digestive tract? What is the nature of the interactions between the different members of the microbiota and the insect host and what are the specific functions performed by the microorganisms, especially in digestion? How do the members of the microbiota interact, or even cooperate, to fulfill functions? And more particularly, what is the ability of *H. illucens* to regulate the microbial community in a decomposing environment? These are fundamental questions that remain largely unanswered.

While it appears that the *H. illucens* digestive tract is an open system capable of hosting a myriad of microorganisms originating from the substrate, whether some of these microbial associates can experience vertical transmission remains unclear. This transmission route is indicative for a stabilized and long-lasting relationship with the host and ensures the transmission of the symbiont and its effects from one generation to the next. The detection of bacterial associates at the egg level raises the question of the existence of such a transmission route, especially for members of the core microbiota. High-throughput sequencing approaches have provided insight into the structure of the *H. illucens* gut microbiota and how it may be influenced by different abiotic and biotic factors. However, these culture-independent approaches have limited resolution in taxonomic identification and provide little access to the deep functional nature of the microbiota. This is why these approaches must work hand in hand with culture-dependent approaches that, integrated in an appropriate workflow, can provide access to the functional dimension of the *H. illucens* gut microbiota, through in silico (genomic analyses), in vitro (phenotyping) and in vivo (immune challenge experiments, etc.) analyses. Metagenomic approaches also allow the capture of a large number of genomes in a single sample. In addition, priority should be given to the study of the biology of the digestive tract of *H. illucens* [39, 110, 112, 172]. A better knowledge of its internal morphology, but also of its functioning is crucial to understand its specific adaptations for digestion and interaction with a wide spectrum of microorganisms, but also to identify new AMPs for potential applications. With the recent sequencing of the *H. illucens* genome [41, 90], an important step has been taken. The annotation of immune and defense genes should shed light on the specific adaptations this insect has evolved to handle the micro-organisms it encounters in its diet and allow transcriptomic approaches to decipher the mechanisms underlying host-microbe interactions in the different compartments of the gut. Finally, the establishment of a more standardized experimental setting, especially for pre-experimental rearing, is desirable for meaningful comparisons between studies. With new data from the *H. illucens* genome and growing collections of microbial associates, there is no doubt that the coming years will be particularly exciting for the study of interactions between *H. illucens* and its microbiota, both at the applied and fundamental levels.

## Data Availability

Not applicable.
